# Distinct requirements for the COMPASS core subunits Set1, Swd1, and Swd3 during meiosis in the budding yeast *Saccharomyces cerevisiae*

**DOI:** 10.1093/g3journal/jkab283

**Published:** 2021-08-05

**Authors:** Brandon M Trainor, Kerri Ciccaglione, Miranda Czymek, Michael J Law

**Affiliations:** 1 Department of Molecular Biology, Graduate School of Biomedical Sciences, Rowan University-School of Osteopathic Medicine, Stratford, NJ 08084, USA; 2 Biochemistry and Molecular Biology Program, School of Natural Sciences and Mathematics, Stockton University, Galloway, NJ 08205, USA; 3 Biology Program, School of Natural Sciences and Mathematics, Stockton University, Galloway, NJ 08205, USA

**Keywords:** histone H3K4 methylation, meiosis, COMPASS complex

## Abstract

Meiosis-specific chromatin structures, guided by histone modifications, are critical mediators of a meiotic transient transcription program and progression through prophase I. Histone H3K4 can be methylated up to three times by the Set1-containing COMPASS complex and each methylation mark corresponds to a different chromatin conformation. The level of H3K4 modification is directed by the activity of additional COMPASS components. In this study, we characterized the role of the COMPASS subunits during meiosis in *Saccharomyces cerevisiae*. In vegetative cells, previous studies revealed a role for subunits Swd2, Sdc1, and Bre2 for H3K4me2 while Spp1 supported trimethylation. However, we found that Bre2 and Sdc1 are required for H3K4me3 as yeast prepare to enter meiosis while Spp1 is not. Interestingly, we identified distinct meiotic functions for the core COMPASS complex members that required for all H3K4me, Set1, Swd1, and Swd3. While Set1 and Swd1 are required for progression through early meiosis, Swd3 is critical for late meiosis and spore morphogenesis. Furthermore, the meiotic requirement for Set1 is independent of H3K4 methylation, suggesting the presence of nonhistone substrates. Finally, checkpoint suppression analyses indicate that Set1 and Swd1 are required for both homologous recombination and chromosome segregation. These data suggest that COMPASS has important new roles for meiosis that are independent of its well-characterized functions during mitotic divisions.

## Introduction

Histone proteins responsible for packaging DNA in the nucleus are subject to an array of post-translational chemical modifications, including acetylation, phosphorylation, and methylation, that are critical regulators of diverse cellular processes. Histone H3Lys4 methylation (H3K4me) is one of the best-studied of the histone modifications. In the budding yeast *Saccharomyces cerevisiae*, all H3K4me is catalyzed by the Set1-containing COMPASS complex (Briggs *et al.* 2001; Roguev *et al.* 2001). COMPASS is an evolutionarily conserved protein complex that is comprised of Set1 and at least six other subunits including Swd1, Swd2, Swd3, Bre2, Sdc1, and Spp1 (Miller *et al.* 2001; Roguev *et al.* 2001; Nagy *et al.* 2002; Santos-Rosa *et al.* 2004). H3K4 can be mono- (me1), di- (me2), or trimethylated (me3) and each methylation level requires specific COMPASS complex members (Schneider *et al.* 2005; Dehe *et al.* 2006). Set1, Swd1, and Swd3 form an enzymatic core that mediates COMPASS complex stability and all H3K4me (Schneider *et al.* 2005; Dehe *et al.* 2006; Mersman *et al.* 2012). In addition, Sdc1, Swd2, and Bre2 are required for H3K4me2 and me3, while Spp1 is important for H3K4me3 (Schneider *et al.* 2005; Dehe *et al.* 2006; Mersman *et al.* 2012). Initial studies of COMPASS function in yeast largely focused on mutations of Set1 that eliminate all H3K4me and, in many cases, uncovered somewhat conflicting results. For example, despite H3K4me3 enrichment at the 5’ ends of actively transcribed genes, Set1 is also required for transcriptional silencing at telomeres and the rDNA locus (Nislow *et al.* 1997; Krogan *et al.* 2002). More recent studies focusing on separate roles for COMPASS subunits during stress response suggested that COMPASS is remodeled to accommodate transcriptional regulation, but the mechanisms underlying this process remain elusive (Margaritis *et al.* 2012; D'Urso *et al.* 2016). Together, these studies indicate that COMPASS-mediated H3K4me is sensitive to growth conditions and regulates cellular processes in a locus-specific manner.

Meiosis is a specialized cell division that produces haploid gametes through one round of DNA replication followed by two rounds of chromosomal division. Meiosis is induced in diploid yeast that is starved for nitrogen and fermentable carbon (Mitchell 1994). Underlying this process is a temporally restricted meiotic transcriptional program that is generally divided into three stages termed early, middle, and late (Chu *et al.* 1998; Primig *et al.* 2000). Post-translational histone modifications regulate the precise timing of gene repression and induction. For example, many early meiotic genes are repressed during vegetative growth by the Ume6 DNA-binding protein, which recruits the Sin3-Rpd3 histone deacetylase to maintain closed chromatin (Strich *et al.* 1994; Kadosh and Struhl 1997). Early gene activation requires Ume6 degradation, which is catalyzed in a two-step mechanism by the histone acetyltransferase Gcn5 (Mallory *et al.* 2007; Mallory *et al.* 2012; Law *et al.* 2014). Similarly, middle meiotic gene repression implicates both histone deacetylation by the Sum1-Rfm1-Hst1 complex and histone H3K4me2 by the COMPASS complex (Xie *et al.* 1999; McCord *et al.* 2003; Jaiswal *et al.* 2017). This repression is relieved upon Sum1 dissociation from chromatin, allowing Ndt80-mediated middle meiotic gene activation and cellular commitment to the meiotic divisions (rev. in Winter 2012). Interestingly, despite being dispensable for vegetative growth and having opposing functions, *sin3*Δ, *rpd3*Δ, *gcn5*Δ, and *set1*Δ mutants arrest early in meiosis (Vidal and Gaber 1991; Vidal *et al.* 1991; Burgess *et al.* 1999; Sollier *et al.* 2004), emphasizing the importance of regulating histone modifications during both meiotic entry and commitment.

While the majority of COMPASS investigations have focused on its roles as a transcriptional regulator, multiple studies have determined that Set1 and Spp1 play transcription-independent roles that are critical for progression through early meiosis (Sommermeyer *et al.* 2013; Jaiswal *et al.* 2017; Adam *et al.* 2018). Following DNA replication, chromosomes are subject to programmed double-stranded DNA breaks (DSBs), homologous chromosome alignment, and genetic recombination. Histone H3K4me3 marks recombination sites by recruiting the Spo11 endonuclease to initiate DSBs at recombination “hotspots” (Keeney *et al.* 1997; Borde *et al.* 2009). Detailed molecular analyses indicate that Spp1 plays COMPASS-independent roles in this process by forming a molecular bridge between H3K4me3 and the DSB machinery (Sommermeyer *et al.* 2013; Adam *et al.* 2018). Interestingly, while both Set1 and H3K4me3 are critical mediators of meiosis, yeast mutants harboring H3K4A point mutations sporulate with near wild-type efficiency (Nislow *et al.* 1997; Sollier *et al.* 2004; Govin *et al.* 2010). These results highlight the complexity of early meiosis and indicate that H3K4me-independent pathways are in place to ensure successful gamete formation. In support of this, yeast *spp1*Δ deletion mutants exhibit meiotic delays that are independent of DSB initiation, indicating redundant pathways are in place to mediate homologous recombination (Adam *et al.* 2018). Finally, while Set1 is a repressor of middle meiotic genes activated by Ndt80, it is required for both Ndt80 expression and progression through meiosis I (MI; Margaritis *et al.* 2012; Jaiswal *et al.* 2017). These data suggest that specific COMPASS complex members are required for different steps in meiosis and may have functions that are independent of their mitotic roles.

Here, we investigated the role of individual COMPASS subunits in the meiotic program in yeast. These studies revealed that *BRE2* and *SDC1* are required for efficient progression through the meiotic program, but are not essential for sporulation. Interestingly, we identified distinct functions for the core COMPASS subunits Set1, Swd1, and Swd3 in executing early and late meiosis, respectively. While Set1 and Swd1 are required for timely progression through prophase I and proper chromosome segregation, Swd3 is important for spore formation. Furthermore, we found that the meiotic requirement of Set1 is independent of H3K4me, suggesting that COMPASS regulates meiotic progression through either a structural role or by targeting nonhistone substrates.

## Materials and methods

### Yeast strains and growth conditions

Genotypes for yeast strains used in this study are listed in Table 1. All strains are in the high-sporulating SK1 genetic background. Homozygous diploid deletion mutants were generated with one-step replacement using the kanamycin (*kanMX6*)- or the hygromycin (*hphMX4*)-resistance marker (Rothstein 1991; Goldstein and McCusker 1999). Transformations were performed using the lithium acetate procedure (Gietz and Woods 2002). Construction of diploid homozygous deletion yeast mutants utilized transformation of haploid deletion mutants with Ycp50-HO. Vegetative yeast was cultured in YEPD (1% Yeast Extract, 2% Peptone, and 1% Dextrose) to a density of ∼5 × 10^7^ cells per ml. Pre-meiotic yeast was cultured in YEPA (1% Yeast Extract, 2% Peptone, and 2% Potassium Acetate) to a density of ∼9 × 10^6^ cells per ml. Meiotic time courses were performed as previously described (Cooper *et al.* 1997). Briefly, yeast was cultured in YEPA to a density of ∼1.2 × 10^7^ cells per ml, harvested, washed twice in ddH_2_O, and resuspended in 1/5th volume of SPII (2% Potassium Acetate, pH = 7.0). Time points were then harvested for subsequent analyses as indicated. All cell densities were determined by light sonication of culture samples followed by microscopic quantification using a hemocytometer.

**Table 1:** Strains used in this study

Strain	Genotype	Source
MLY1	*MAT* ***a*** */MATα lys2/lys2 trp1::hisG/trp1::hisG ura3/ura3 LYS2::ho*Δ*/LYS2::ho*Δ	(Strich *et al.* 1994)
MLY20	*MAT* ***a*** */MATα lys2/lys2 trp1::hisG/trp1::hisG ura3/ura3 LYS2::ho*Δ*/LYS2:: ho*Δ *SET1-9myc-TRP1/SET1-9-myc-TRP1*	This study
MLY86	*MAT* ***a*** */MATα lys2/lys2 trp1::hisG/trp1::hisG ura3/ura3 LYS2::ho*Δ*/LYS2::ho*Δ *set1:: KanMX/set1::KanMX*	(Law and Ciccaglione 2015)
MLY234	*MAT* ***a*** */MATα lys2/lys2 trp1::hisG/trp1::hisG ura3/ura3 LYS2::ho*Δ*/LYS2::ho*Δ *swd3::HphMX/swd3::HphMX*	This study
MLY270	*MAT* ***a*** */MATα lys2/lys2 trp1::hisG/trp1::hisG ura3/ura3 LYS2::ho*Δ*/LYS2::ho*Δ *spp1::HphMX/spp1::HphMX*	This study
MLY338	*MAT* ***a*** */MATα lys2/lys2 trp1::hisG/trp1::hisG ura3/ura3 LYS2::ho*Δ*/LYS2::ho*Δ *HHT1::KanMX/HHT1::KanMX HHT2-K4A/HHT2-K4A*	This study
MLY372	*MAT* ***a*** */MATα lys2/lys2 trp1::hisG/trp1::hisG ura3/ura3 LYS2::ho*Δ*/LYS2::ho*Δ *sdc1:: KanMX/sdc1::KanMX*	This study
MLY373	*MAT* ***a*** */MATα lys2/lys2 trp1::hisG/trp1::hisG ura3/ura3 LYS2::ho*Δ*/LYS2::ho*Δ *swd1::KanMX/swd1::KanMX*	This study
MLY374	*MAT* ***a*** */MATα lys2/lys2 trp1::hisG/trp1::hisG ura3/ura3 LYS2::ho*Δ*/LYS2::ho*Δ *bre2:: KanMX/bre2::KanMX*	This study
MLY385	*MAT* ***a*** */MATα lys2/lys2 trp1::hisG/trp1::hisG ura3/ura3 LYS2::ho*Δ*/LYS2:: ho*Δ *SET1-9myc-TRP1/SET1-9-myc-TRP1 swd1::KanMX/swd1::KanMX*	This study
MLY386	*MAT* ***a*** */MATα lys2/lys2 trp1::hisG/trp1::hisG ura3/ura3 LYS2::ho*Δ*/LYS2:: ho*Δ *SET1-9myc-TRP1/SET1-9-myc-TRP1 swd3::KanMX/swd3::KanMX*	This study
MLY544	*MAT* ***a*** */MATα lys2/lys2 trp1::hisG/trp1::hisG ura3/ura3 LYS2::ho*Δ*/LYS2::ho*Δ *set1 ::KanMX/set1::KanMX swd3::HphMX/swd3::HphMX*	This study
MLY585	*MAT* ***a*** */MATα lys2/lys2 trp1::hisG/trp1::hisG ura3/ura3 LYS2::ho*Δ*/LYS2::ho*Δ *set1 ::HphMX/set1::HphMX mad2::KanMX/mad2::KanMX*	This study
MLY614	*MAT* ***a*** */MATα lys2/lys2 trp1::hisG/trp1::hisG ura3/ura3 LYS2::ho*Δ*/LYS2::ho*Δ *set1 ::HphMX/set1::HphMX rad9::KanMX/rad9::KanMX*	This study
MLY621	*MAT* ***a*** */MATα lys2/lys2 trp1::hisG/trp1::hisG ura3/ura3 LYS2::ho*Δ*/LYS2::ho*Δ *swd1::HphMX/swd1::HphMX rad9::KanMX/rad9::KanMX*	This study
MLY622	*MAT* ***a*** */MATα lys2/lys2 trp1::hisG/trp1::hisG ura3/ura3 LYS2::ho*Δ*/LYS2::ho*Δ *swd1::HphMX/swd1::HphMX mad2::KanMX/mad2::KanMX*	This study
MLY634	*MAT* ***a*** */MATα lys2/lys2 trp1::hisG/trp1::hisG ura3/ura3 LYS2::ho*Δ*/LYS2::ho*Δ *set1 ::HphMX/set1::HphMX pch2::KanMX/pch2::KanMX*	This study
MLY635	*MAT* ***a*** */MATα lys2/lys2 trp1::hisG/trp1::hisG ura3/ura3 LYS2::ho*Δ*/LYS2::ho*Δ *swd1::HphMX/swd1::HphMX pch2::KanMX/pch2::KanMX*	This study
MLY641	*MAT* ***a*** */MATα lys2/lys2 trp1::hisG/trp1::hisG ura3/ura3 LYS2::ho*Δ*/LYS2::ho*Δ *set1:: HphMX/set1::HphMX spo11::KanMX/spo11::KanMX*	This study
MLY642	*MAT* ***a*** */MATα lys2/lys2 trp1::hisG/trp1::hisG ura3/ura3 LYS2::ho*Δ*/LYS2::ho*Δ *swd1::HphMX/swd1::HphMX spo11::KanMX/spo11::KanMX*	
MLY646	*MAT* ***a*** */MATα lys2/lys2 trp1::hisG/trp1::hisG ura3/ura3 LYS2::ho*Δ*/LYS2::ho*Δ *HHT1::KanMX/HHT1::KanMX HHT2-K4A/HHT2-K4A set1::HphMX/set1::HphMX*	This study

### Meiotic phenotype analyses

#### Terminal meiotic phenotypes

Meiotic completion was determined by quantifying the spore percentage of triplicate cultures 24 h after meiotic induction using bright-field microscopy. For each experiment, at least 200 cells were counted.

#### Spore viability analyses

Spore viability was scored by macroscopic colony formation on rich medium following tetrad dissection as previously described (Amberg *et al.* 2005). Ascospore wall digestion was accomplished using zymolyase (5 U per µl, Zymo Research) treatment for 5–10 min at room temperature. Viability of at least 80 spores per strain was quantified. Aneuploidy was confirmed using PCR analyses of the mating-type locus *HO* as previously described (White and Haber 1990).

#### Meiotic progression

Meiotic progression was monitored by fixing cells in 70% ethanol then staining with 4’,6-diaminidino-2-phenylindole (DAPI) as described (Pringle *et al.* 1989). At least 200 cells per time point were counted to monitor progression through MI (bi-nucleated cells) and MII (3–4 nucleated cells). Quantification of DAPI-staining cells for each single deletion mutant was performed for the first 12 h of the time course and a final sample was analyzed after 24 h in SPM. Double deletion mutants were assayed for meiotic progression after 12- and 24-h in SPM.

### Microscopy

Bright field and fluorescence microscopy were performed using a Leica DM4000B microscope equipped with a Leica DFC450 C digital CCD camera. Calcofluor White and Eosin Y staining was performed on cells that were sporulated in liquid SPM for 24 h as previously described (Lin *et al.* 2013). Cells were first harvested and washed in 1 ml McIlvaine’s buffer (0.2 M Na_2_HPO_4_/0.1 M citric acid, pH = 6.0). Staining was then performed using 30 µl Eosin Y disodium salt (5 mg/ml, Sigma) in 500 µl McIlvaine’s buffer for 10 min at room temperature in the dark. Cells were washed twice in McIlvaine’s buffer and resuspended in 200 µl McIlvaine’s buffer containing 1 µl of 1 mg/ml Calcofluor White solution (Sigma). Fluorescence of Calcofluor White and Eosin Y was examined using the DAPI and FITC filters, respectively. At least 200 cells were counted for the presence of internalized Eosin Y staining.

### Ether resistance assay

Ether resistance was assayed essentially as previously described with the following modifications (Lin *et al.* 2013). Cells were sporulated for 24 h in liquid SPM, harvested, and resuspended in sterile ddH_2_O. Cell densities were then equilibrated and either resuspended in sterile ddH_2_O or ether for 2 min of exposure. 10-fold serial dilutions of ether-treated and -untreated samples were then spotted onto YEPD agar, incubated at 30°C for 3 days and the images were collected.

### Western blot analysis

Protein extracts were prepared from vegetative, pre-meiotic, or meiotic cultures as described previously (Cooper *et al.* 1997). Either 100 or 25 µg of whole-cell protein extract was used for α-myc-Set1 or histone H3 modifications, respectively. Following transfer onto PVDF, membranes were incubated with α-H3 C-terminal domain (1:5000; Abcam ab1791), α-H3K4me1 (1:2500; Abcam ab8895), α-H3K4me2 (1:2500; Abcam ab11946), or α-H3K4me3 (1:2500; Abcam ab8580). Myc-epitope tagged Set1 protein levels were monitored using α-myc (1:3000; Abcam ab32) with α-alpha Tubulin (1:5000; Abcam ab184970) or α-Pgk1 (1:5000; Abcam ab113687) serving as a loading control. Secondary antibodies were conjugated with alkaline phosphatase (1:5000; α-mouse Abcam ab6790; α-rabbit Abcam ab97097) and signal was detected using CDP star detection reagent.

### RT-qPCR

Total nucleic acids were prepared from 2 ml of sporulation culture using Trizol (Thermo Fisher) and mechanical lysis with glass beads according to the manufacturer’s instructions. Approximately 1 µg of total nucleic acid preparations were treated with DNase I (New England Biolabs) and then reverse transcribed using ProtoScript II reverse transcriptase (New England Biolabs) in oligo-dT primed reactions according to the manufacturer’s instructions. Subsequent qPCR reactions were prepared using the Power SYBR Master mix (Applied Biosystems) containing primers listed in Table 2. All *C_T_* values were normalized first to *ACT1*, then to wild-type values at *t* = 0 (ΔΔ*C_T_*). Values reported are the average of three or more independent biological replicates; error bars represent the standard error of the mean.

**Table 2:** qPCR primers used in this study

Primer	Sequence (5’ -> 3’)
*IME1* cod f	TCC CCT AGA AGT TGG CAT TTT G
*IME1* cod r	CCA AGT TCT GCA GCT GAG ATG A
*IME2* cod f	AAT GTT TTG GGT GAT GCC TCT T
*IME2* cod r	TTC TTG GAG TAA AAT CTG GCA TTG
*NDT80* cod f	GCG CTA GGT GCA CCG AAC T
*NDT80* cod r	CAT TGG TGT GGA TTG ACG AGA T
*ACT1* cod f	TCG TTC CAA TTT ACG CTG GTT
*ACT1* cod r	CGG CCA ATC GAT TCT CAA

## Results

### The COMPASS complex and H3K4me are sensitive to pre-meiotic growth conditions

**Figure 1:**
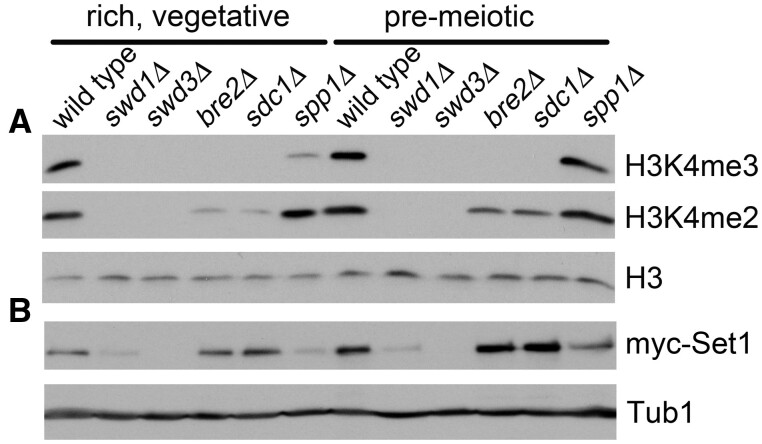
COMPASS subunit requirements for H3K4me catalysis and Set1 stability are sensitive to pre-meiotic growth conditions. Western blot analyses for wild-type or yeast harboring COMPASS deletion mutations grown to mid-logarithmic phase in rich, vegetative, or pre-meiotic growth conditions were performed. Membranes were incubated with (A) anti-H3K4me3, anti-H3K4me2, or (B) anti-myc epitope antibodies with anti-H3 C-terminal domain or anti-Tub1 antibodies serving as loading controls.

Commitment to meiosis is a step-wise process that begins as cells are cultured in pre-meiotic growth conditions. Multiple lines of evidence support this, including (1) transcription of the Inducer of Meiosis (*IME1*) is elevated, (2) mitotic cyclins, whose function is replaced by the meiosis-specific factor *IME2*, are downregulated, (3) Ume6 protein, a major repressor of early meiotic genes whose degradation is required for meiotic entry, is decreased by 50%, and (4) H3K4me3 patterns associated with meiotic recombination sites are established (Mai and Breeden 2000; Mallory *et al.* 2007; Borde *et al.* 2009; Strudwick *et al.* 2010). To determine the contribution of specific COMPASS subunits in maintaining H3K4me in pre-meiotic growth conditions, we performed Western blot analyses measuring H3K4me2 or me3 in wild-type or yeast mutants lacking individual COMPASS complex members. We chose not to evaluate the impact of deleting *SWD2* in this investigation, due to its essential contributions as part of the cleavage and polyadenylation factor complex (Roguev *et al.* 2001; Cheng *et al.* 2004; Dichtl *et al.* 2004). These experiments were performed on yeast cultured in either vegetative or pre-meiotic conditions (see Materials and Methods). Wild-type cells display similar levels of H3K4me2 and me3 regardless of growth condition, suggesting that global COMPASS complex activity was intact (Figure 1A). Consistent with previous reports, *SWD1* and *SWD3* are required for H3K4me2 and me3 in vegetative conditions (Dehe *et al.* 2006; Mersman *et al.* 2012; Kim *et al.* 2013) and this requirement is maintained in pre-meiotic growth conditions, indicating that both Swd1 and Swd3 are essential for COMPASS-mediated H3K4me (Figure 1A). We found that H3K4me2 levels are greatly diminished for *bre2*Δ and *sdc1*Δ mutants cultured in vegetative conditions, which agrees well with other reports (Figure 1A; Dehe *et al.* 2006; Mersman *et al.* 2012; Kim *et al.* 2013). In contrast, we observed more subtle defects in H3K4me2 for these mutants during pre-meiotic growth, suggesting a growth condition-dependent alteration in COMPASS behavior (Figure 1A). Finally, while *SPP1* is required for maximal H3K4me3 levels in vegetative growth conditions, it is dispensable for this modification during pre-meiotic growth (Figure 1A). This finding indicates that COMPASS can maintain H3K4me3 in pre-meiotic growth conditions independently of Spp1. Together, these data suggest that the requirement for Bre2, Sdc1, and Spp1 for H3K4me2 and me3 is sensitive to pre-meiotic growth conditions.

Previous studies have determined that Set1 protein levels are undetectable in the absence of *SWD1* and *SWD3* and are greatly diminished in *spp1*Δ mutants (Dehe *et al.* 2006; Mersman *et al.* 2012). To determine the requirement of COMPASS subunits for maintaining Set1 protein levels in pre-meiotic growth conditions, we performed Western blot analyses measuring myc-epitope tagged Set1 in wild-type or COMPASS deletion yeast mutants. As described above, these experiments were performed on yeast cultured to mid-logarithmic phase in either vegetative or pre-meiotic cultures. We observed moderately increased Set1 protein levels in wild-type yeast cultured in pre-meiotic growth conditions as compared to rich, vegetative cultures (Figure 1B). This is in contrast to similar levels of H3K4me2 and me3 observed across both growth conditions (Figure 1A). Consistent with other reports, we found that Set1 protein levels are drastically decreased in vegetative cultures for *swd1*Δ, *swd3*Δ, and *spp1*Δ mutants (Figure 1B, left panel; Dehe *et al.* 2006; Mersman *et al.* 2012). Interestingly, we observed a modest increase in Set1 protein levels for *bre2*Δ, *sdc1*Δ, and *spp1*Δ mutant yeast in pre-meiotic cultures relative to vegetative growth conditions (Figure 1B). This observation is similar to the increased Set1 protein levels for wild-type, pre-meiotic yeast cultures, suggesting that Set1 expression is sensitive to culture conditions. Despite these increases in Set1 protein, yeast lacking *BRE2* or *SDC1* are unable to catalyze H3K4me3 (Figure 1A). This is in contrast to *SPP1*, which is dispensable for H3K4me3 in pre-meiotic cultures (Figure 1A). Together, these data suggest that *BRE2* and *SDC1* are required for H3K4me3 in pre-meiotic growth conditions, while *SPP1* is not. This is consistent with previous work indicating that Spp1 plays COMPASS-independent functions during stress response and in early meiosis (D'Urso *et al.* 2016; Adam *et al.* 2018). Finally, we found that *SWD1* and *SWD3* are required for maintaining Set1 protein levels, regardless of the growth condition (Figure 1B). These results indicate that Set1 protein levels are increased as yeast are preparing to enter meiosis and support a model in which some of the well-characterized interactions between COMPASS complex subunits that occur during vegetative growth may be altered to accommodate meiosis.

### COMPASS complex subunits play separate roles in meiosis

**Figure 2:**
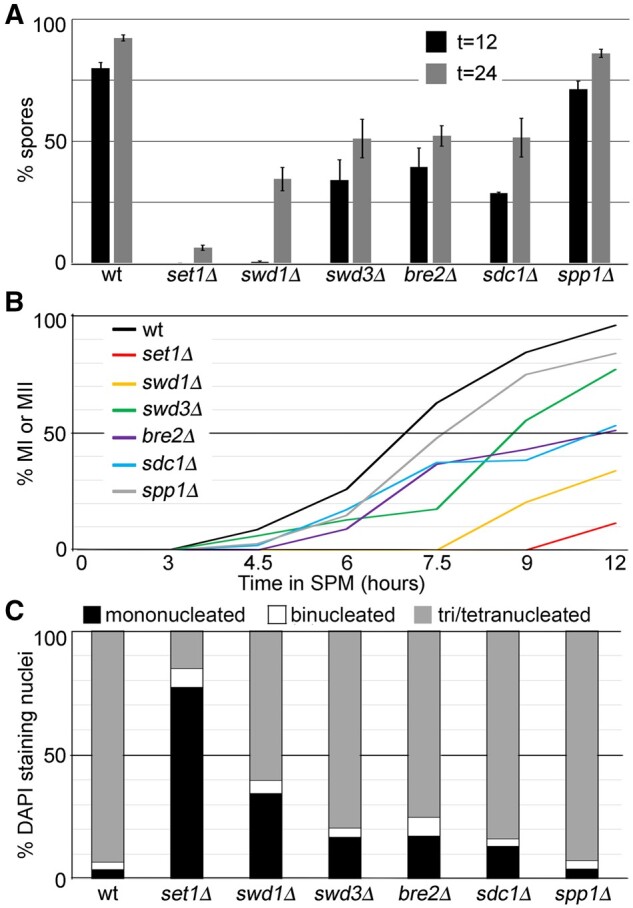
Distinct roles for COMPASS subunits during meiosis. Terminal meiotic phenotypes and meiotic progression were monitored in wild-type or yeast harboring COMPASS deletion mutations. (A) Spore percentages were quantified 12 or 24 h after meiotic induction using bright field microscopy. Graphs depict the average for three independent biological replicates; error bars show the standard error of the mean. (B) Kinetics of meiotic divisions were monitored by quantifying the number of DAPI staining nuclei per cell. Time points were harvested as indicated and the number of cells containing 2 or 4 nuclei are shown. A representative time course experiment is shown. (C) Quantification of DAPI staining nuclei for yeast harboring the indicated deletion mutations was performed 24 h after meiotic induction.

We found that COMPASS subunits had distinct roles during pre-meiotic growth. Therefore, we next tested whether this was also the case during meiosis. To address this question, we performed time-course experiments for wild type and COMPASS deletion mutants measuring their progression through, and successful completion of, meiosis (see Materials and Methods for details). While spore percentages allow determination of a cell’s ability to progress through meiosis and form microscopically visible ascospores, they do not provide an indication of the kinetics of meiosis in individual genotypes. Similarly, cells that fail to form spores might have arrested at any point in the meiotic program prior to this final step. Therefore, we quantify DAPI staining bodies in individual cells to indicate progression through the MI and MII divisions, thus allowing us to discriminate between mutants that fail to form spores but have progressed through one or both meiotic divisions. Finally, cells might form ascospores containing aneuploid spores due to mistakes in chromosome segregation, which can be determined using measurements of spore viability. Consistent with previous reports, wild-type and *spp1*Δ mutants complete meiosis (Figure 2A) with similar kinetics (Figure 2B) and produce viable gametes (Table 3; Acquaviva *et al.* 2013; Sommermeyer *et al.* 2013). In contrast, we observed *bre2*Δ and *sdc1*Δ single mutants display an ∼50% reduction in spore formation after 24 h in SPM (Figure 2A). Despite a delay in meiotic divisions, >80% of *bre2*Δ or *sdc1*Δ mutants have completed both MI and MII prior to 24 h in SPM, suggesting that *BRE2* and *SDC1* are important for efficient completion of meiosis, but are not essential for meiosis (Figure 2, B and C; Supplementary Figure S1). In support of this, we observed 97.5% viable spores in *sdc1*Δ mutants, while *bre2*Δ mutants showed a mild reduction to 67.5% viability (Table 3). Finally, we observed disparate roles for the core COMPASS complex members *SET1*, *SWD1*, and *SWD3* during meiosis. First, *SET1* is required for efficient sporulation and meiotic divisions as <10% of the *set1*Δ mutants form spores and ∼15% of these mutants have completed MI and MII after 24 h in SPM (Figure 2). Interestingly, the *set1*Δ mutants that are able to complete meiosis form viable spores, which agrees well with previous investigations (Sollier *et al.* 2004) and indicates compensatory mechanisms are in place to ensure meiosis occurs without functional Set1 (Table 3). Second, *SWD1* is required for efficient sporulation by 12 h; however, *swd1*Δ mutants exhibit delayed, but not absent sporulation (Figure 2). In contrast to *set1*Δ mutants, *swd1*Δ mutants have 42.5% viable spores, suggesting that Swd1 is important for meiotic chromosome segregation (Table 3). Finally, *swd3*Δ mutants display delays in MI and MII and reduced spore numbers that are more similar to those observed in *bre2*Δ and *sdc1*Δ mutants than *set1*Δ or *swd1*Δ mutations (Figure 2). Importantly, spore viability in *swd3*Δ mutants is moderately impaired, indicating that Swd3 is less important for chromosome segregation than Swd1 (Table 3). These observations are in stark contrast to previous studies performed in multiple labs indicating that Set1, Swd1, and Swd3 have overlapping requirements for H3K4 methylation and chromosome behavior (Krogan *et al.* 2002; Schneider *et al.* 2005; Dehe *et al.* 2006; South *et al.* 2010; Mersman *et al.* 2012; Soares *et al.* 2014; Thornton *et al.* 2014). The remainder of this study focuses on further characterizing the roles of these core COMPASS complex members during yeast meiosis.

**Table 3:** Spore viability for compass deletion mutant strains

Strain genotype	% viable spores	Number of spores analyzed
Wild type	97.5	240
*set1*Δ	100	80
*swd1*Δ	42.5	240
*swd3*Δ	70.4	240
*bre2*Δ	67.5	120
*sdc1*Δ	97.5	120
*spp1*Δ	88.4	120
H3K4A	96.3	120

### SET1, but not histone H3K4 methylation, is required for meiosis

**Figure 3:**
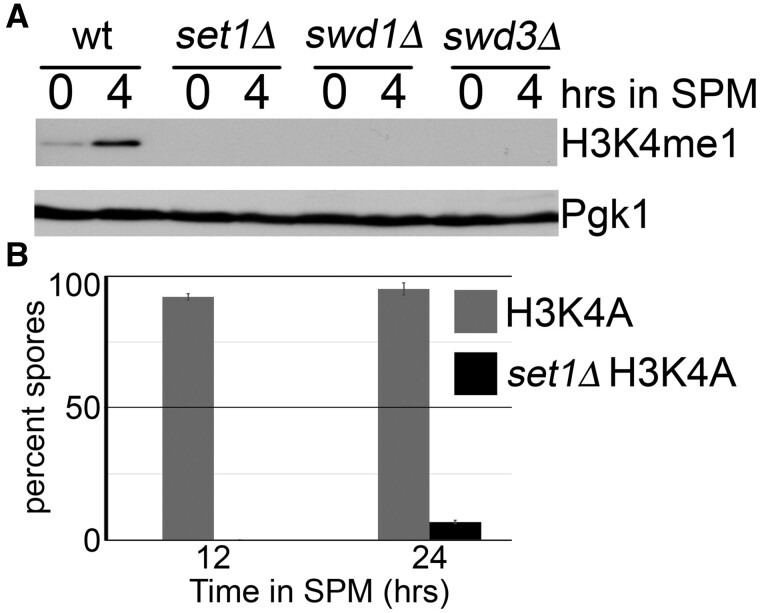
Meiotic phenotypes for the COMPASS core complex are independent of histone H3K4me. (A) The COMPASS core complex is required for H3K4me1 during meiosis. Western blot analyses measuring histone H3K4me1 for wild-type or yeast mutants harboring the indicated deletion mutations. Total protein extracts were prepared from yeast grown in pre-meiotic conditions or 4 h post-meiotic induction. Membranes were incubated with anti-H3K4me1 antibody with anti-Pgk1 serving as the loading control. (B) H3K4me is dispensable for meiosis. Yeast harboring chromosomally integrated histone H3K4A point mutations with wild-type or *SET1* deletion mutations were induced to enter meiosis. Graphs represent the average spore percentage for three independent biological replicates; error bars show the standard error of the mean.

Along with Set1, Swd1 and Swd3 form an enzymatic core for the COMPASS complex that is essential for all H3K4me and Set1 protein stability (Krogan *et al.* 2002; Schneider *et al.* 2005; Dehe *et al.* 2006; South *et al.* 2010; Mersman *et al.* 2012; Soares *et al.* 2014; Thornton *et al.* 2014). To determine if the differences in Swd1- and Swd3-dependent meiosis are related to histone H3K4me catalysis, we performed Western blot analyses measuring H3K4me1 levels in wild type, *set1*Δ*, swd1*Δ, or *swd3*Δ yeast mutants during meiosis (Figure 3A). We decided to focus on H3K4me1 as this modification is a prerequisite for subsequent H3K4me2 and me3 marks (Schneider *et al.* 2005; Dehe *et al.* 2006; Kim *et al.* 2013). As cells enter meiosis, wild-type yeast exhibit low levels of H3K4me1 that increase 4 h into the meiotic program (Figure 3A). Importantly, Set1, Swd1, and Swd3 are all required for H3K4me1 in meiosis, indicating that the differences we observe for these COMPASS members during meiosis are independent of their requirement for H3K4me catalysis.

The lack of measurable H3K4me1 in *swd3*Δ mutants combined with their ability to form viable gametes raised the possibility that H3K4me is dispensable for meiosis. To test this possibility, we measured both sporulation percentage and spore viability of a yeast mutant harboring the chromosomally integrated H3K4A mutation (Figure 3B and Table 3). Consistent with previous investigations (Govin *et al.* 2010), we found that H3K4 is dispensable for spore formation and viability, suggesting that the requirement of Set1 for meiosis is independent of H3K4me. To test this possibility, we performed meiotic analyses in a H3K4A *set1*Δ mutant. Interestingly, we found that *SET1* is required for meiosis even when methylation of histone H3K4 is not possible (Figure 3B), suggesting that Set1 methylates a nonhistone substrate or plays a structural role in coordinating progress through meiotic prophase.

### SET1 and SWD1 are required for meiotic transcriptional timing

Meiosis and spore morphogenesis require a transient transcriptional program generally divided into three stages termed early, middle, and late. Early meiotic genes, induced by the master regulator of meiosis *IME1*, are responsible for initiating meiotic DNA replication and homologous recombination (Kassir *et al.* 1988; Smith and Mitchell 1989; rev. in Winter 2012). Middle gene expression, coordinated by the meiosis-specific transcription factor Ndt80, results in commitment to the meiotic divisions and initiates enclosure of each haploid nucleus in the prospore membrane (Chu and Herskowitz 1998; Hepworth *et al.* 1998; Pak and Segall 2002; Sopko *et al.* 2002). Late genes stimulate spore maturation, chromatin compaction, and spore wall assembly, allowing the development of a mature ascus (Neiman 2011). Misregulating early meiotic gene transcription can result in meiotic prophase arrest, preventing Ndt80 activation, middle meiotic gene expression and chromosome division (Chu and Herskowitz 1998; Hepworth *et al.* 1998; Tung *et al.* 2000). In contrast, errors in middle or late meiosis permit chromosome segregation while causing specific defects in spore wall assembly (Neiman 1998; Suda *et al.* 2009; Neiman 2011; Lin *et al.* 2013).

**Figure 4:**
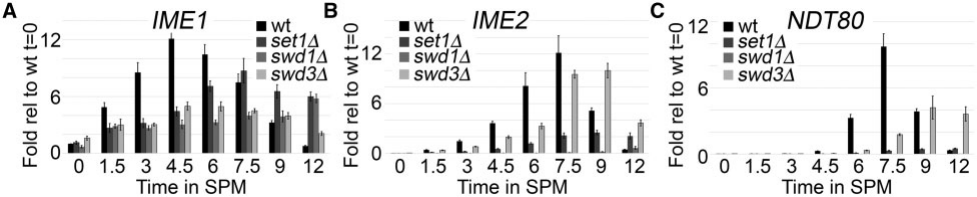
*SET1* and *SWD1* are required for the meiotic transcriptional cascade. RT-qPCR analyses for wild-type or yeast mutants harboring COMPASS core deletion mutations was performed during a meiotic time-course experiment. Total RNA was prepared from biological triplicate yeast meiotic cultures that were harvested at the indicated time points. Following reverse transcription, qPCR reactions were performed using primers that amplify (A) *IME1*, (B) *IME2*, or (C) *NDT80*. Target abundance was normalized to *ACT1* levels and reported values are relative to wild-type expression at *t* = 0. The relative values for *IME2* are × 10^2^ and *NDT80* × 10^2^.

To determine the requirement of the COMPASS core for executing this program we performed meiotic time course experiments and measured gene expression using RT-qPCR. Our analyses centered on the expression of *IME1*, *IME2*, and *NDT80*, three genes that play central roles in meiotic progression. Expression of the master regulator of meiosis *IME1* peaks in wild-type yeast at 4.5 h and decreases through the remainder of the time course (Figure 4A). *IME1* expression was delayed by ∼3 h in *set1*Δ mutants suggesting that, despite their failure to perform MI and MII, *SET1* is not required for the initiation of meiosis (Figure 4A). Both *swd1*Δ and *swd3*Δ mutants have reduced expression of *IME1*; however, *IME1* expression peaks in *swd1*Δ mutants at 12 h, while the pattern of *IME1* expression in *swd3*Δ mutants is more similar to wild-type cells (Figure 4A). This indicates that while both Swd1 and Swd3 are required for maximal *IME1* expression, Swd1 is more important for the timing of *IME1* induction. Together, these data suggest that the meiotic defects observed in COMPASS core mutants are not due to a failure to induce the meiotic program through *IME1* expression, but might be caused by reduced *IME1* expression levels.

We next measured the transcription of *IME2*, an early meiotic gene that encodes a protein kinase required for progression through meiotic prophase (rev. in Winter 2012). Wild-type yeast induce *IME2* 1.5 h into meiosis and expression peaks at 7.5 h (Figure 4B). Yeast lacking *SET1* have greatly reduced, but not absent, *IME2* expression (Figure 4B). In contrast, *SWD1* is required for *IME2* expression, suggesting that Set1 and Swd1 may play different roles in early meiosis (Figure 4B). Finally, *SWD3* is necessary for the proper kinetics of *IME2* expression, consistent with its role in the timing of MI and MII.

*NDT80* expression is stimulated by Ime2 and represents a key event in the cellular commitment to the meiotic divisions. Wild-type yeast induce low-level *NDT80* expression at 4.5 h, consistent with their progression through MI at this time point (Figures 2B and 4C). Similar to our observations for *IME2* expression, we observed delayed and reduced *NDT80* in *swd3*Δ mutants, supporting its role in efficient meiotic timing and completion (Figure 4C). Finally, we found that *SET1* and *SWD1* are required for *NDT80* expression, which agrees well with their requirement for progression through MI and MII (Figure 4C). Together, these data suggest that *SET1* and *SWD1* are required for distinct steps during early meiosis that lead to meiotic divisions, while *SWD3* is more important for the appropriate timing of meiotic transcription and commitment to form gametes.

### SET1 and SWD1 are required for meiotic prophase and spindle assembly

**Figure 5:**
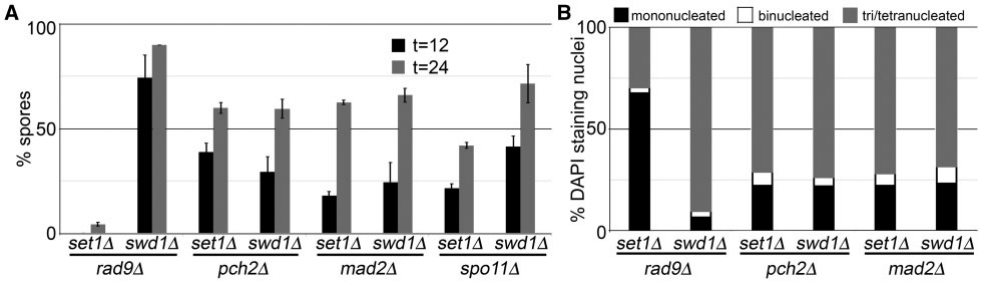
*SET1* and *SWD1* are required for different steps of meiotic commitment. (A) Spore percentages and (B) DAPI staining bodies were quantified for yeast harboring deletion mutations in *SET1* or *SWD1* combined with the checkpoint genes *RAD9*, *PCH2*, or *MAD2*. (A) Spore percentages are reported 12 and 24 h post-meiotic induction and include deletion mutations for the *SPO11* endonuclease. (B) DAPI staining nuclei were quantified following 24 h in SPM. Graphs represent the average spore percentage for independent biological triplicates; error bars depict the standard error of the mean.

Our observations indicate that *SET1* and *SWD1* are required for *NDT80* expression and that this requirement may occur via two different mechanisms. Yeast lacking *SET1* allow low-level induction of *IME2* whereas *swd1*Δ mutants fail to induce *IME2*. These data could be explained by locus-specific differences in H3K4me, leading to misregulation of gene expression. However, since H3K4 is dispensable for sporulation, this possibility is eliminated. Alternatively, the observed deficiencies in the meiotic transcription program could be due to a requirement for *SET1* and *SWD1* to progress past early meiotic checkpoints. To test this possibility, we performed meiotic analyses of yeast lacking *SET1* or *SWD1* in combination with deletion mutations of three checkpoint-related genes *RAD9*, *PCH2*, or *MAD2*. Rad9 will arrest meiotic cells that harbor chromosomal lesions after pre-meiotic S-phase but prior to recombination and synapsis (Weber and Byers 1992). Previous studies have demonstrated that yeast harboring *rad9*Δ mutations have no measurable defects in meiotic progression, homologous recombination frequency, spore formation, or spore viability (Weber and Byers 1992). Interestingly, yeast harboring *set1*Δ*rad9*Δ mutations fail to sporulate, but *swd1*Δ*rad9*Δ mutants sporulate with ∼90% efficiency (Figure 5A). Quantification of DAPI staining nuclei in the *set1*Δ*rad9*Δ mutants indicated an approximately twofold increase in progression through MI and MII relative to *set1*Δ mutants alone, suggesting a role for *RAD9* in suppressing the *set1*Δ-associated meiotic phenotype (Figures 5B and 2C). Both the *set1*Δ*rad9*Δ and *swd1*Δ*rad9*Δ mutants have reduced spore viability, indicating improper chromosome segregation (Table 4). These results suggest that Swd1 either prevents DNA damage during replication or is important for post-replicative DNA repair, but Set1 does not.

**Table 4:** Spore viability for checkpoint double mutants

Strain genotype	% viable spores	Number of spores analyzed
*set1*Δ*rad9*Δ	32.5	80
*swd1*Δ*rad9*Δ	40	120
*set1*Δ*pch2*Δ	57.5	120
*swd1*Δ*pch2*Δ	35	120
*set1*Δ*mad2*Δ	47.5	120
*swd1*Δ*mad2*Δ	17.5	120
*set1*Δ*spo11*Δ	8.4	120
*swd1*Δ*spo11*Δ	0	120

The pachytene checkpoint ensures that chromosome segregation is inhibited in the presence of meiotic recombination or chromosome synapsis errors (rev in. Roeder and Bailis 2000). Pch2 is a meiosis-specific component of the pachytene checkpoint pathway that monitors synaptonemal complex (SC) formation and is dispensable for normal homologous recombination, chromosome segregation, and sporulation (San-Segundo and Roeder 1999). We examined the requirement for *PCH2* in the meiotic arrest of *set1*Δ and *swd1*Δ yeast mutants by testing the ability of *set1*Δ*pch2*Δ and *swd1*Δ*pch2*Δ mutants to form viable spores. After 12 h in sporulation medium, we found that *set1*Δ*pch2*Δ mutants produce 38% spores while *swd1*Δ*pch2*Δ mutants produce 30% spores (Figure 5A). These spore percentages increase for both strains after 24 h of sporulation. These data indicate a partial requirement for *PCH2* in mediating *set1*Δ and *swd1*Δ mutant meiotic arrest. In support of this conclusion, we found that ∼20% of *set1*Δ*pch2*Δ and *swd1*Δ*pch2*Δ mutants remain mononucleated after 24 h in sporulation conditions (Figure 5B). Interestingly, despite increased sporulation efficiency, both *set1*Δ*pch2*Δ and *swd1*Δ*pch2*Δ mutants have reduced spore viability, indicating defects in chromosome segregation in these mutants (Table 4).

The suppression of *set1*Δ and *swd1*Δ mutant meiotic arrest by *pch2*Δ mutations suggests that Set1 and Swd1 mediate SC formation. To determine if this is an indirect result of impaired DSB formation, we performed meiotic analyses in yeast lacking the meiosis-specific endonuclease Spo11. Previous studies indicated that *SET1* is required for the normal distribution of Spo11-induced DSBs, but it is not essential for DSB formation (Sollier *et al.* 2004; Borde *et al.* 2009). Yeast lacking *SPO11* fail to initiate DSBs and bypass the recombination and chromosome synapsis checkpoints (Klapholz *et al.* 1985; Keeney *et al.* 1997). To determine if *SPO11* is required for the *set1*Δ and *swd1*Δ meiotic arrest phenotype, we performed meiotic analyses of *set1*Δ*spo11*Δ and *swd1*Δ*spo11*Δ mutants (Figure 5). These experiments revealed that ∼25% of *set1*Δ*spo11*Δ mutants form spores after 12 h in sporulation medium, and this number increases to ∼40% of after 24 h (Figure 5). These data suggest that the *set1*Δ meiotic arrest is partially dependent on Spo11-mediated DSB formation. We observed a similar, but more robust, rescue of sporulation in *swd1*Δ*spo11*Δ mutants with ∼40% spores after 12 h of growth in sporulation medium that increases to >70% sporulation after 24 h (Figure 5). These data suggest that DSB formation plays a more significant role in the meiotic arrest observed in *swd1*Δ mutants. In both cases, microdissection of the resulting spores revealed that they are inviable, consistent with previous investigations indicating that *SPO11* is required for meiotic chromosome segregation (Klapholz *et al.* 1985; Wagstaff *et al.* 1985; Cao *et al.* 1990). Together, these data are consistent with a role in homologous recombination for both Set1 and Swd1.

Finally, we investigated the role of the spindle assembly checkpoint (SAC) in mediating *SET1*- and *SWD1-*dependent meiotic arrest. Similar to mitosis, chromosome segregation during meiosis requires proper spindle assembly and attachment to kinetochores (Li and Nicklas 1995; Biggins and Murray 2001). The SAC ensures that these steps are faithfully completed to allow proper chromosome segregation and avoid aneuploidy. The SAC component Mad2 is important for proper meiotic chromosome segregation and regulates the timing of cellular progression through MI (Shonn *et al.* 2003; Tsuchiya *et al.* 2011). Importantly, previous investigations of mitotic chromosome division demonstrated that Set1-mediated H3K4me negatively regulates Mad2 until the spindle attaches to chromosomes (Schibler *et al.* 2016). To determine if the absence of *SET1* and *SWD1* lead to hyperactivation of Mad2 and meiotic arrest, we performed meiotic analyses of *set1*Δ*mad2*Δ and *swd1*Δ*mad2*Δ mutants. Similar to our analyses of *PCH2*, we found that *set1*Δ*mad2*Δ and *swd1*Δ*mad2*Δ mutants produce ∼20% spores after 12 h in sporulation medium and that the spore percentages increase to ∼65% at the 24-h time point (Figure 5A). Consistent with a partial role for *MAD2* in *SET1*- and *SWD1-*dependent meiotic progression, 25% of *set1*Δ*mad2*Δ and *swd1*Δ*mad2*Δ mutants remain mononucleated after 24 h in sporulation medium (Figure 5B). Microdissection of the resulting asci revealed defects in spore viability, as 47.5% of *set1*Δ *mad2*Δ mutant spores were viable and only 17.5% of *swd1*Δ*mad2*Δ mutant spores survived (Table 4). The reduced spore viability in the *set1*Δ*mad2*Δ and *swd1*Δ*mad2*Δ yeast mutants also reflects the high rates of nondisjunction observed when Mad2 is removed in isolation (Shonn *et al.*^2000^, ^2003^). Our results suggest that both Set1 and Swd1 are important for spindle attachment to the kinetochore during the meiotic divisions. Together, our data support a model in which both Set1 and Swd1 are critical for the faithful execution of multiple steps during prophase I, including recombination and spindle assembly. Furthermore, Swd1 displays a Set1-independent role in regulating replicative chromosomal lesions, as supported by the Rad9-dependent arrest in *swd1*Δ mutants (Figure 5A).

### SWD3 is involved in late spore morphogenesis

**Figure 6:**
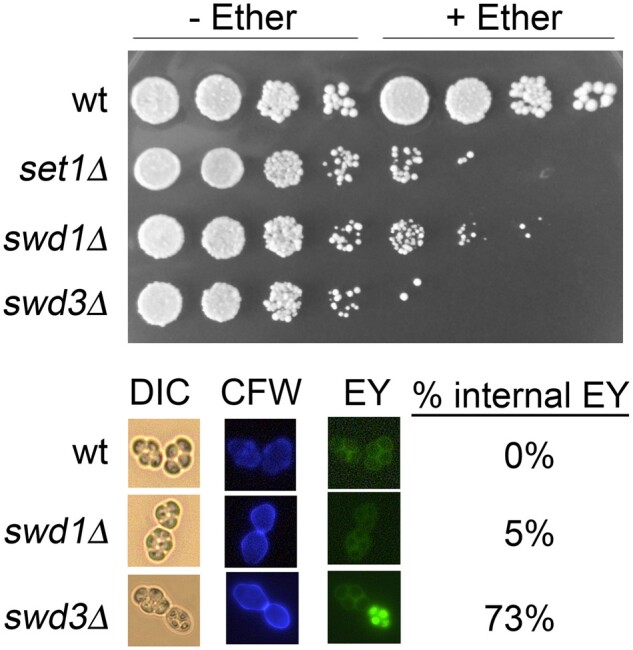
*SWD3* supports late spore morphogenesis and spore resistance to stress. (A) Strains with the indicated genotypes were sporulated for 24 h in SPM media. Cells were harvested, washed in ddH_2_O, and resuspended in ether. 10-fold serial dilutions of treated and untreated cells were spotted onto YEPD plates. (B) Cells of the indicated genotypes were sporulated and stained with both Calcofluor White (CFW) and Eosin Y (EY). At least 200 cells were examined to quantify the Eosin Y staining patterns.

Following the completion of MII, yeast undergo morphogenic pathway directing prospore membrane formation and spore wall assembly thus protecting the newly formed haploid gametes from environmental stressors (Briza *et al.* 1990). Spore wall assembly occurs sequentially in a process that first deposits mannan and beta-glucan, whose orientation is reversed relative to vegetative cells, and the spore-specific chitosan and dityrosine layers (Kreger-Van Rij 1978; Briza *et al.* 1988; Smits *et al.* 2001). Importantly, spore wall formation is dependent upon both the expression of Ndt80-dependent middle and late meiotic genes and the activity of Smk1 and Ssp2 protein kinases (Chu *et al.* 1998; Chu and Herskowitz 1998; Primig *et al.* 2000; Omerza *et al.* 2018). Spore sensitivity to ether is a frequently used assay that allows measurement of spore wall assembly. While ether is toxic to vegetative cells, yeast gametes with properly formed spore walls are resistant to ether exposure (Dawes and Hardie 1974). To determine if COMPASS is required for spore wall formation, we performed ether sensitivity assays on the COMPASS deletion mutants (Figure 6A and Supplementary Figure S2). Consistent with previous reports, we found that wild-type yeast spores are resistant to ether exposure (Figure 6A). Both *set1*Δ and *swd1*Δ cause increased ether sensitivity, however, both of these strains also have reduced spore numbers. For example, while *set1*Δ mutant spores are ∼100-fold more sensitive to ether than wild-type spores, the mutant strains have a 10-fold reduction in total spore numbers (Figures 2A and 6A). Similarly, *swd1*Δ mutants display ∼10–100-fold increases in ether sensitivity yet sporulate with approximately threefold reduced rates relative to wild type (Figures 6A and 2A). In contrast, *swd3*Δ mutants form approximately twofold fewer spores than wild-type, these deletion mutants are >1000-fold more sensitive to ether exposure (Figures 2A and 6A). These data suggest that *SWD3* is important for proper spore wall formation.

To further characterize the role of Swd3 in spore wall assembly, we performed fluorescence microscopy of wild-type, *swd1*Δ and *swd3*Δ mutant spores stained with both Calcofluor white and Eosin Y. Calcofluor white is a chitin/chitosan stain that can only access spores if there is a defective dityrosine layer, while Eosin Y will specifically stain chitosan. We eliminated *set1*Δ mutants from these analyses due to the low spore numbers in this mutant (Figure 2A). In all strains analyzed, we observed minimal Calcofluor white staining indicating that the dityrosine layer is intact in these spores (Figure 6B;Tachikawa *et al.* 2001). Consistent with previous reports, Eosin Y staining of wild-type cells revealed a chitosan layer surrounding each individual spore (Figure 6B; Baker *et al.* 2007; Lin *et al.* 2013). Interestingly, while we observed robust Eosin Y staining in both the *swd1*Δ and *swd3*Δ mutants, the distribution of the stain showed a unique pattern (Figure 6B). In these mutant strains, an aberrant Eosin Y staining pattern was visible inside of individual spores. Quantification of this irregular staining distribution revealed that ∼5% of *swd1*Δ mutant spores contain Eosin Y staining that is internalized, while ∼73% of *swd3*Δ mutant spores have this staining pattern (Figure 6B). Furthermore, the internalized Eosin Y staining pattern appeared to be random, as the same ascus could contain anywhere from 1 to 4 spores displaying this unique spore morphology. Together, these data suggest that the increased ether sensitivity observed for *swd3*Δ mutants may be due to improper chitosan distribution in the spore wall, as opposed to failures in chitosan production.

## Discussion

This study reveals new roles for the core COMPASS complex members Set1, Swd1, and Swd3 in executing meiosis. Our findings are consistent with a model in which both Set1 and Swd1 promote progression through meiotic prophase by acting at multiple execution points. Checkpoint suppression analyses indicated that Set1 is required for homologous recombination and meiotic spindle assembly. These results are consistent with previous reports suggesting that Set1 is required for efficient meiotic DSB distribution and mitotic spindle assembly (Sollier *et al.* 2004; Borde *et al.* 2009; Schibler *et al.* 2016). Interestingly, our results indicate that the role for Set1 during prophase is independent of H3K4me, suggesting the presence of nonhistone substrates or an important structural role for the COMPASS complex during meiosis. In contrast to Set1, Swd1 appears to act earlier in the meiotic program and is involved in the post-replicative repair pathway. Finally, we find that Swd3 is dispensable for meiotic prophase, but plays an important role during late meiosis. Specifically, yeast mutants lacking Swd3 are hypersensitive to ether exposure and display errors in ascospore wall assembly. Together, these results indicate that Set1, Swd1, and Swd3 have previously undescribed roles in efficient meiotic timing and gametogenesis that are independent of their well-characterized functions for catalyzing H3K4me catalysis.

### Meiosis-specific functions for COMPASS subunits

In contrast to the well-studied functions for COMPASS during vegetative growth, investigations focused on meiosis have been more limited (Borde *et al.* 2000; Sollier *et al.* 2004; Borde *et al.* 2009; Sommermeyer *et al.* 2013; Adam *et al.* 2018). Similar to other histone-modifying complexes, Set1 is dispensable for mitotic divisions, but is required for meiosis (Sollier *et al.* 2004; Borde *et al.* 2009; Jaiswal *et al.* 2017). This highlights the sensitivity of the temporally regulated meiotic program to the balance in histone modification complex activity. In addition to targeting histone substrates, this investigation and others indicate that modifications of nonhistone proteins are also critical regulators of meiosis (Mallory *et al.* 2012; Law *et al.* 2014). Furthermore, our results suggest that meiosis-specific COMPASS functions are a key aspect of meiotic timing and commitment. In support of this, previous studies have found that Spp1 stimulates H3K4me3 by interacting with the COMPASS complex during vegetative growth. As cells enter meiotic prophase, Spp1 plays COMPASS-independent roles to coordinate DSBs by forming a molecular bridge spanning both H3K4me3 and the chromosomes axis protein Mer2 (Acquaviva *et al.* 2013; Sommermeyer *et al.* 2013; Adam *et al.* 2018). Interestingly, despite the importance of both H3K4me3 and Spp1 to efficient DSB formation, yeast mutants lacking either Set1 or Spp1 form re-distributed DSBs at reduced levels (Borde *et al.* 2009). These data indicate that multiple compensatory mechanisms are in place to ensure that genetic recombination occurs during meiosis, thus resulting in genetically diverse haploid gametes. Identifying other factors that direct Spo11 to initiate DSBs will be an important step to resolving this key step in gametogenesis.

Similar to Spp1, the results presented here are consistent with meiosis-specific functions for Swd1 and Swd3. While most studies have indicated that Swd1 and Swd3 are genetically and biochemically inseparable during vegetative growth, transcriptomic analyses indicate minor differences in their requirement for gene repression (Margaritis *et al.* 2012). In contrast to this subtle distinction, our results indicate dramatically different requirements for Swd1 and Swd3 in early and late meiosis respectively. Interestingly, our data suggest that Swd1 and Swd3 function during meiosis is independent of their roles in catalyzing H3K4me as part of the COMPASS complex.

These results are consistent with at least two models that describe the requirement of COMPASS for meiosis. In the first model, COMPASS catalyzes methylation of nonhistone substrates whose modification is required for meiosis. The only nonhistone substrate for COMPASS identified to date is Dam1, a component of a heterodecameric protein complex that comprises the kinetochore (Cheeseman *et al.* 2001; Janke *et al.* 2002; Li *et al.* 2002; Westermann *et al.* 2005; Zhang *et al.* 2005). Interactions between the kinetochore and microtubules stimulate sister chromatid separation during both mitosis and MII and therefore must be modified to accommodate homologous chromosome separation during MI (Marston and Amon 2004). During MI kinetochores of sister chromatids are co-oriented and attach to microtubules from the same spindle pole utilizing a protein complex termed monopolin (Toth *et al.* 2000). Interestingly, the timing of microtubule-kinetochore attachments appears to be a critical regulator of the chromosome divisions during meiosis. For example, inhibition of these attachments through ectopic expression of the meiosis-specific factor Mam1 during mitosis results in MI-like reductional divisions (Miller *et al.* 2012). Conversely, inducing microtubule-kinetochore attachments prematurely during MI results in sister chromatid segregation as observed during mitosis (Miller *et al.* 2012). Therefore, one model to explain the H3K4me-independent functions of COMPASS during meiosis implicates Dam1 methylation as a key regulator of microtubule attachments during MI. While a precise role for Dam1 methylation has not yet been identified, previous studies indicate that methylation inhibits phosphorylation of neighboring serines by the Aurora kinase Ipl1 and is important for a “methyl-phospho” switch (Zhang *et al.* 2005). Interestingly, kinetochore attachment to microtubules requires Dam1 phosphorylation, suggesting that failure to methylate Dam1 may allow premature phosphorylation and cause defects in chromosome segregation during MI. This is supported by our findings of aneuploidy in gametes formed from *set1*Δ and *set1*Δ yeast mutants harboring deletions in the Mad2 SAC protein. Interestingly, while previous work found that both Set1 and Swd1 are required for Dam1 methylation, the role of Swd3 in catalyzing this modification was not reported (Latham *et al.* 2011). This raises the possibility that Dam1 methylation occurs independently of Swd3, thus resulting in the disparate requirements for Set1, Swd1, and Swd3 during MI.

A second model that could explain our results involves noncatalytic, structural roles for COMPASS in orchestrating progression through meiotic prophase. Recent, high-resolution structural studies of the COMPASS complex indicate the presence of extensive interactions with the nucleosome (Worden *et al.* 2020). For example, Set1/Bre2 interact directly with nucleosomal DNA on one arm of the complex while Swd1/Spp1 bind to DNA on the opposite arm (Worden *et al.* 2020). This establishes a COMPASS/nucleosome interface that positions both Swd1 and Set1 in close proximity to the histone core. Point mutations of key basic residues in Swd1 that are responsible for contacting DNA cause a moderate reduction in H3K4me2 and me3, indicating that maintaining these interactions is a key component of catalytic activity (Worden *et al.* 2020). In contrast to Set1 and Swd1, Swd3 appears away from DNA on the backside of the COMPASS complex and does not contribute to direct interactions between COMPASS and the nucleosome (Worden *et al.* 2020). These data suggest that eliminating either Set1 or Swd1 would cause dissociation of the COMPASS complex from nucleosomes, perhaps influencing local chromatin structure independently of methyltransferase activity. It is intriguing that *swd3*Δ mutants cause destabilization of Set1, yet do not phenocopy *set1*Δ mutants during meiosis. This observation raises questions about locus-specific COMPASS assemblies and their ability to maintain local chromatin structures via both catalytic and structural mechanisms. Future experiments aimed at discriminating between a structural or catalytic role for COMPASS during meiosis will be a crucial next step in determining how this highly conserved protein complex regulates gametogenesis. In support of this, separation of function alleles for the Set1 homolog *MLL3/4* revealed that methyltransferase activity is less critical for embryonic stem cell development than its structural role (rev. in Morgan and Shilatifard 2020). Importantly, these studies first demonstrated that the mutant *MLL3/4* alleles allow wild-type protein expression and retain interactions with their binding partners. Similar analyses of catalytically inactive Set1 in yeast have either destabilized Set1 or have not directly examined interactions with COMPASS complex subunits (Williamson *et al.* 2013; Soares *et al.* 2014). Furthermore, different catalytically inactive Set1 alleles do not phenocopy one another, indicating that the mutant proteins have off-target effects that impact cellular behavior (Williamson *et al.* 2013). Comprehensive analyses of these various mutants will be critically important to separating the catalytic role of COMPASS during meiosis versus its structural function.

### Genetic interactions between COMPASS and HORMA-domain proteins

Our results and others indicate that COMPASS genetically interacts with proteins that harbor a common protein domain termed HORMA (Hop1, Rev7, Mad2; Aravind and Koonin 1998; Schibler *et al.* 2016). HORMA domain-containing proteins are required for coordinating meiotic prophase and are characterized by an N-terminal core domain and a C-terminal “safety belt” (Rosenberg and Corbett 2015). The safety belt region interacts with the HORMA core domain in two distinct conformations termed “open” and “closed,” which in turn mediates protein-protein interactions. HORMA proteins interact with binding partners in the “closed” state and these interactions are critical for their meiotic functions. How these conformational changes are regulated throughout meiotic prophase remains poorly understood.

Some insight into how COMPASS regulates HORMA proteins can be gleaned from the checkpoint suppression analyses reported in this study. First, we found that the requirement for *SET1* and *SWD1* during meiotic prophase is suppressed by mutation of *PCH2*. Pch2 is a conserved protein that mediates Hop1 removal following successful SC formation (San-Segundo and Roeder 1999). Hop1 interacts with Spo11 via the linker protein Red1 and these interactions are critical for normal DSB formation (Smith and Roeder 1997; de los Santos and Hollingsworth 1999; Panizza *et al.* 2011). Our data suggest that in the absence of *SET1* or *SWD1*, Hop1 is unable to establish the SC, leading to Pch2-mediated meiotic arrest. This is consistent with the requirement of Set1 for DSB distribution (Sollier *et al.* 2004; Sommermeyer *et al.* 2013) and is further supported by our findings that *spo11*Δ suppresses *set1*Δ and *swd1*Δ meiotic defects.

Second, we found that deletion of the HORMA protein Mad2 suppresses *set1*Δ and *swd1*Δ mutant meiotic arrest. Mad2 is a component of the SAC that monitors microtubule-kinetochore attachments during chromosome divisions in both mitosis and meiosis (Li and Murray 1991; Pesin and Orr-Weaver 2008; Lau and Murray 2012; Jia *et al.* 2013). The SAC is deactivated when microtubules properly attach to the kinetochore and establish tension, permitting Anaphase Promoting Complex/Cyclosome (APC/C) activation (Jia *et al.* 2013). The APC/C is an E3 ubiquitin ligase that targets protein substrates, such as Pds1, for degradation by the 26S proteasome (Jia *et al.* 2013). Both APC/C activation and substrate specificity are mediated by auxiliary factors such as Cdc20 and Cdh1 and the meiosis-specific protein Ama1 (Dawson *et al.* 1995; Visintin *et al.* 1997; Cooper *et al.* 2000). Previous work indicates that H3K4me inactivates the SAC during mitosis by directly interacting with Mad2 and separating it from APC/C^Cdc20^ (Schibler *et al.* 2016). This interaction relieves APC/C^Cdc20^ repression, allowing cells to progress through anaphase. In this model, COMPASS restricts Cdc20-mediated proteolysis until the proper conditions for chromosome separation have been met. Our results partially support a similar role for H3K4me in regulating Mad2 activity and progression through anaphase I of meiosis. For example, our data indicate that the subunits required for H3K4me2, *BRE2* and *SDC1*, are important for both meiotic timing and completion (Figure 2). Furthermore, we found that both *set1*Δ*mad2*Δ and *swd1*Δ*mad2*Δ mutants progress past meiosis I, but have increased aneuploidy (Figure 5 and Table 4). These data suggest both Set1 and Swd1 are important for proper chromosome segregation during meiosis, consistent with a role in spindle attachment to the kinetochore. This points to important roles for COMPASS mediated methylation of both histone H3K4 and nonhistone substrates in regulating meiotic chromosome segregation.

Finally, our data indicate that Swd1 is involved in Rad9-dependent post-replicative DNA damage repair during meiosis. One mechanism that cells can utilize to bypass DNA lesions involves error-prone translesion DNA synthesis by the DNA Polymerase Zeta complex, which contains the HORMA protein Rev7 (Polζ; Nelson *et al.* 1996; Andersen *et al.* 2008). Yeast mutants lacking Rad9 display a Rev7-dependent hypermutability phenotype, indicating that Rad9 antagonizes the error-prone repair pathway in favor of an error-free one (Murakami-Sekimata *et al.* 2010). In addition to its well-characterized role in translesion DNA synthesis, Rev7 is also implicated in meiotic DSB processing. Intriguingly, recent reports indicate Spo11-induced DSBs during meiosis are associated with Polζ-mediated repair mechanisms (Rattray *et al.* 2015). The net result of this DNA repair is an increased mutation rate at DSB hotspots, which increases the genetic variation in the haploid gametes. These results suggest that Swd1 may be an important regulatory factor in directing Rev7 activity to repair DSBs during meiosis. Identifying how changes in both COMPASS composition and locus-specific activity contribute to genetic recombination during meiosis will be an important question in the future.

## Data availability

Strains are available upon request. The authors affirm that all data necessary for confirming the conclusions of the article are present within the article, figures, and tables.

Supplementary material is available at G3 online.

## Supplementary Material

jkab283_Supplementary_Data
