# Supplementary figures and images for: Distinct requirements for the COMPASS core subunits Set1, Swd1, and Swd3 during meiosis in the budding yeast Saccharomyces cerevisiae
Source: G3 (Bethesda). 2021 Aug 5;11(11):jkab283. doi: 10.1093/g3journal/jkab283 (PMC8527496; doi:10.1093/g3journal/jkab283)

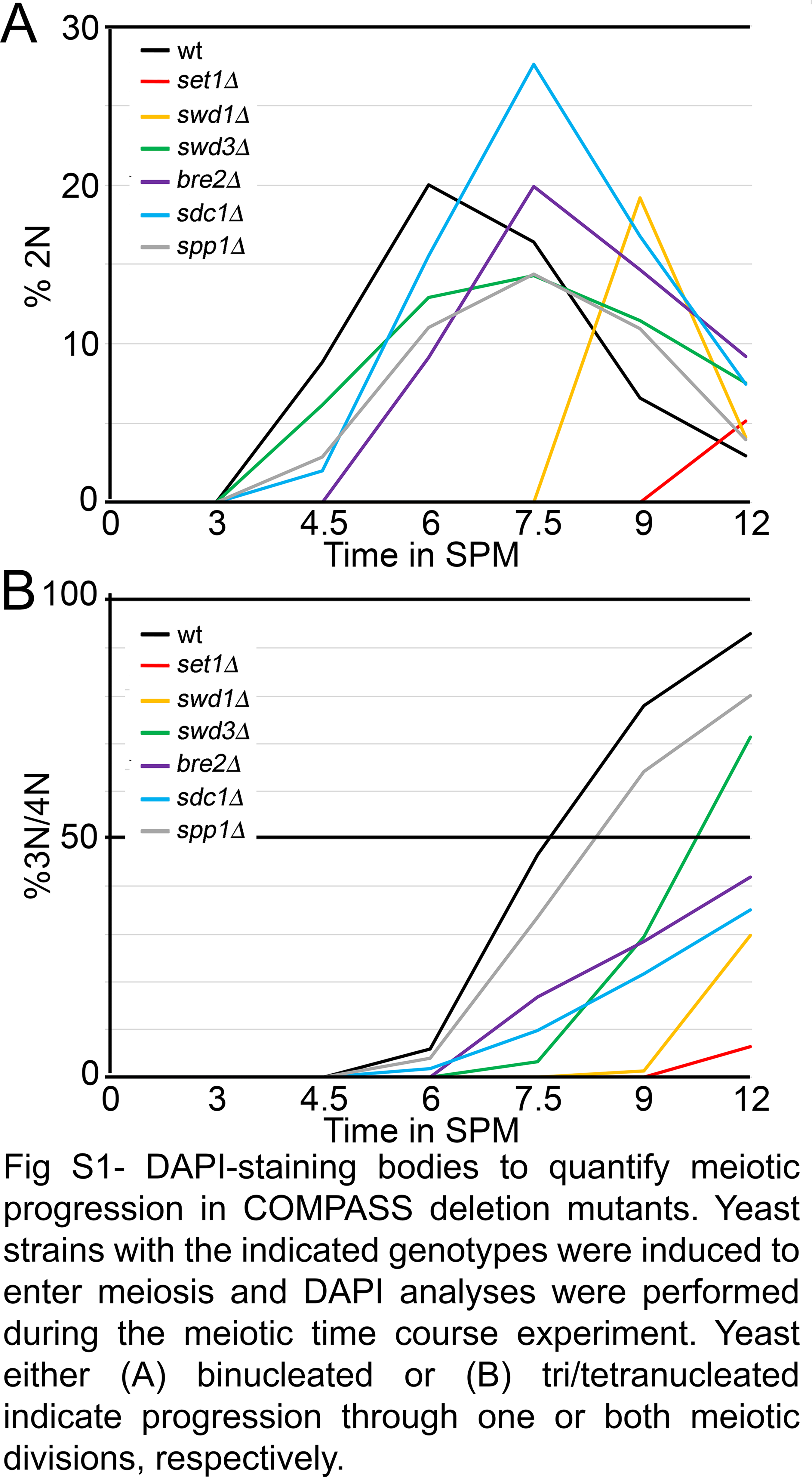

Supplement: jkab283_Supplementary_Data [file jkab283_supplementary_data.zip › GENETICS-G3-2021-402738-s01.tif]

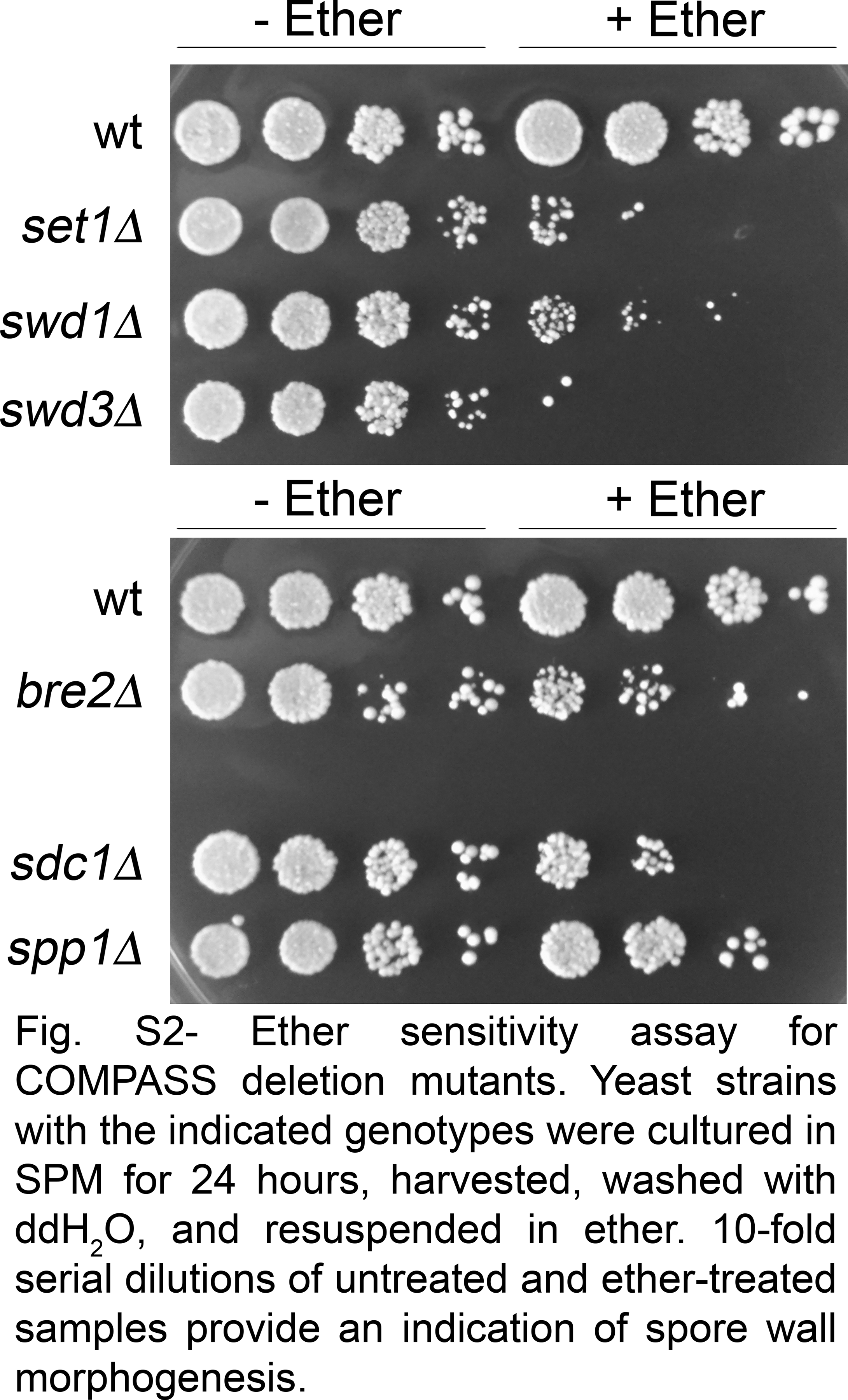

Supplement: jkab283_Supplementary_Data [file jkab283_supplementary_data.zip › GENETICS-G3-2021-402738-s02.tif]
